# Effect of subclinical hypothyroidism on the skeletal system and improvement with short-term thyroxine therapy

**DOI:** 10.18632/oncotarget.19568

**Published:** 2017-07-26

**Authors:** Cuixia Gao, Yu Wang, Tingting Li, Jing Huang, Limin Tian

**Affiliations:** ^1^ Department of Ultrasonic Diagnosis, Gansu Provincial Hospital, Lanzhou, China; ^2^ Department of Endocrinology, Gansu Provincial Hospital, Lanzhou, China

**Keywords:** subclinical hypothyroidism, bone, L-thyroxine

## Abstract

The purpose of the study was to observe changes in the skeletal system of rats with subclinical hypothyroidism (SCH) and to determine whether L-thyroxine (L-T4) administration suppresses those changes. Sixty male Wistar rats were randomly divided into control, SCH, and SCH+T4 groups. SCH was induced in rats by administration of methimazole (MMI), and rats in the SCH+T4 group were treated with L-T4 after 45 days of MMI administration. The SCH group had higher thyroid-stimulating hormone (TSH) level than the control and SCH+T4 groups. There were no differences in serum thyroid hormone (FT4 and FT3) levels among the three groups. Bone mineral density; serum levels of BALP and TRACP-5b, two bone metabolic markers; and the biomechanical properties of the femurs were lower in the SCH group than in the control group. After L-T4 treatment, serum BALP and TRACP-5b levels and the femur biomechanical properties were higher in the SCH+T4 than the SCH group. Histopathological examination revealed damage to the structure of the femur trabecular bone network in rats with SCH, and L-T4 treatment improved this condition to some extent. These findings demonstrate that L-T4 treatment ameliorates the destructive effects of SCH on the skeletal system in rats.

## INTRODUCTION

Subclinical hypothyroidism (SCH) is an increased concentration of thyroid-stimulating hormone (TSH) with a normal level of thyroid hormone [1]. SCH, the mildest form of hypothyroidism, lacks the specific signs and symptoms of clinical hypothyroidism [2]. The prevalence of SCH is between 4% and 20% in the general population and higher in women than in men, ranging from 11% to 17% in community-dwelling elderly populations [3–6]. SCH progresses to overt hypothyroidism in approximately 2% to 5% of cases annually [7]. Overt thyroid dysfunction is associated with decreased bone mineral density (BMD) and increased risk of fracture [8, 9]. Thyroid hormone is a factor in bone remodeling that acts directly or indirectly on bone cells, influencing bone resorption and bone formation [10, 11].

Before the report of Abe et al. [12], TSH was known to stimulate the development of thyroid follicles and release thyroid hormone [13]. Osteoporosis related to thyroid dysfunction was considered to be the result of the changes in thyroid hormones [14]. However, Abe et al. [12] reported that TSH itself inhibits bone remodeling by binding to the TSH receptor (TSHR) on the osteoblast and osteoclast precursors, and it directly disrupts bone metabolism independent of thyroid hormones. TSHR knockout (TSHR−/−) mice with congenital hypothyroidism display high-turnover osteoporosis. Although the T4 level is normal, heterozygous TSHR+/− mice have decreased bone mineral density (BMD). This finding suggests an increase in TSH levels might promote bone metabolism. Previous studies have demonstrated the lower TSH level is correlated with lower BMD and increased risk of osteoporotic fractures [15–17]. A cross-sectional study [18] indicated that women with SCH have reduced femoral neck BMD, and another study showed that older men with SCH are at increased risk for hip fracture [19]. However, other reports found different results [20, 21]. Thus, the effect of SCH on the skeletal system remains unclear. We chose the animal experiment that more effectively excludes the interference factors from the study of the effect of SCH on the skeletal system. The aim of the present study was to investigate the effects of SCH on the skeletal system, including BMD, bone metabolism, biomechanical properties, and bone microstructure, in addition to determining whether administration of L-thyroxine (L-T4) improves the osteoporosis risk profile.

## MATERIALS AND METHODS

### Animal model

A total of 60 male SPF Wistar rats (provided by Gansu University of Chinese Medicine Experimental Animal Center, Lanzhou, China) weighing 140 g to 160 g were randomly divided into three groups: the control euthyroid group (C, *n* = 20), the subclinical hypothyroid group (SCH, *n* = 20), and the subclinical hypothyroid group treated with L-T4 (SCH+T4, *n* = 20). All the rats were fed in the specific pathogen free (SPF) animal laboratory (Gansu University of Chinese Medicine Experimental Animal Center, Lanzhou, China). SCH was induced in the rats by administration of 10 mg/kg/d of methimazole (MMI) by gavage for 3 months. The dose of MMI in our study was determined on the basis of previous studies. These studies established the hypothyroid rat model, so we reduced the dose by fivefold to establish the SCH rat model. After 45 days of MMI administration, we continued MMI administration (10 mg/kg) once daily by gavage to the rats but only added a daily intragastric injection of L-T4 at the dose of 2 μg/kg/d in the SCH+L-T4 group for 45 days. The control rats received physiological saline via gavage. During the 3-month period, body weight was measured daily. Serum thyroid hormone and TSH levels were measured every 2 weeks to verify the success of the induction of SCH in rats.

### Drugs

All the drugs used in the experiments were obtained commercially from Sigma (USA) and were freshly prepared in Krebs-bicarbonate solution. MMI and L-T4 were dissolved in physiological saline.

### Thyroid function test

Fasting blood samples of all rats were collected from the tail vein every 2 weeks after an overnight fast of at least 8 hours and centrifuged 10 minutes. Serum samples for biochemical analyses were collected and stored at −20°C. FT4, FT3, and TSH serum concentrations were measured by use of ELISA kits (Yuanye Biotechnology, Shanghai, China).

### Bone metabolism factors measurements

Fasting blood of all rats was collected from the arteria femoralis. Serum samples were collected by centrifugation and stored at −20°C until analysis. The serum concentrations of bone-specific alkaline phosphatase (BALP) and tartrate-resistant acid phosphatase 5b (TRACP-5b) were measured by use of ELISA kits (Fengxiang Biotechnology, Shanghai, China). Serum C-terminal telopeptides of type I collagen (CTX) were measured by use of ELISA kits (Cloud-Clone Co., Ltd., Wuhan, China). Serum calcium (Ca) was measured by an Arsenazo 2I method utilizing a Calcium Assay Kit (Beijing Strong Biotechnologies, Beijing, China). Serum phosphorus (P) was measured by the phosphomolybdate reduction method (Beijing Strong Biotechnologies, Beijing, China).

### Bone mass measurements

All rats were euthanized after blood samples were collected from the arteria femoralis. BMD in skull, spine, upper limbs, lower extremities, trunk, ribs, pelvis, and whole body of all rats were measured by dual-energy x-ray absorptiometry (DEXA) (MEDILINK, Osteocore-3, France) from the Lanzhou Army General Hospital utilizing small-animal software (Hologic, Inc.). The equipment was calibrated according to the manufacturer's instructions. The same investigator analyzed all scans.

### Biomechanical examination

The frozen left femora in saline-soaked gauze was thawed in physiological saline solution at room temperature for 1 hour before the three-point bending test. The biomechanical properties of the femora were evaluated by use of a commercial mechanical testing device (AGS-10kNG; Shimadzu, Kyoto, Japan). The samples were submerged in physiological saline solution until undergoing mechanical testing. The femur with its physiological curvature facing up was stabilized on a sample supporter with two fixed loading points with a 17-mm interval distance. The upper loading plate was oriented perpendicularly to the long axis of the femur, and the upper loading point was located at the midpoint between the two lower loading points. A preload of 2N was applied to immobilize the sample before the mechanical testing. Load was applied at a constant displacement rate of 2 mm/min by controlling the motion of the upper loading plate until bone fracture occurred. The load-displacement curve was simultaneously plotted and data were automatically recorded by a computer that was interfaced with the material-testing machine. The following parameters were directly obtained from the load-displacement curve: maximum load (maximum force that the femur can sustain before failure), stiffness (slope of the linear part of the load-displacement curve representing elastic deformation), and energy absorption (area under the load-deformation curve until failure). Elastic modulus was calculated according to the formula E~FL3 = 48 dI, where F is the maximum load, L is the distance between the two lower supporting points, d is the displacement, and I is the moment of inertia of the cross-section in relation to the horizontal axis of the femur.

### Bone histology

The right femurs of rats in the three groups were placed in 4% paraformaldehyde, fixed 2 to 3 days, and then decalcified in 0.5 M EDTA (pH = 8.0) for 45 days. The right proximal femurs were embedded in paraffin by standard histological procedures. Sections 5 μm in thickness were cut and stained with hematoxylin & eosin (H&E, 40×) and visualized under the light microscope (Olympus BX 53).

### Ethics statement

This study was performed in strict accordance with the recommendations of the Guide for the Care and Use of Laboratory Animals by the National Institutes of Health. The protocol was approved by the Committee on the Ethics of Animal Experiments of the Gansu University of Chinese Medicine Experimental Animal Center (Permit Number: SYXK 2011–0001).

### Statistics

The values are presented as the mean ± standard deviation. One-way analysis of variance (ANOVA) was performed to compare the changes of data among the SCH, SCH+T4, and control groups. The least significant difference test was used to identify the differences. All statistical analyses already described were conducted by SPSS 21 software (SPSS Inc., Chicago, IL, USA). *P* values <0.05 were statistically significant.

## RESULTS

### Morphological variables

**Figure 1:**
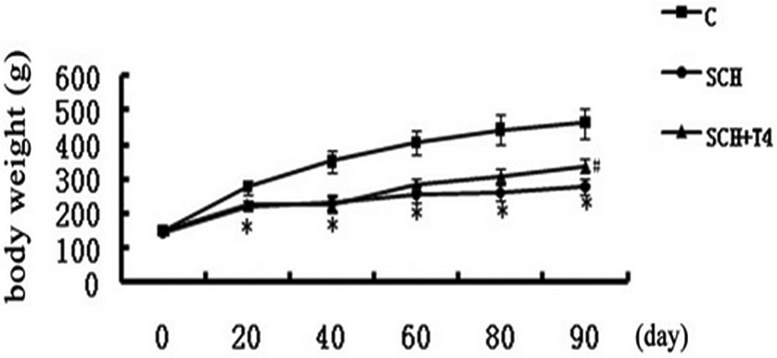
Body weights of rats in three groups *P < 0.05 versus control group; ^#^*P* < 0.05 versus SCH group.

Compared with rats in control group, rats with SCH experienced stunted growth during the 3-month study. The rats with SCH were less active and had dryer fur than the control rats, and body weights were significantly lower in the SCH group than in the control group (Figure 1). These results were similar to those of previous studies [22–25]. At the end of the 3-month study period, we found that body weights increased in the SCH+T4 treatment groups (Figure 1). The physiological symptoms were also ameliorated in the SCH+T4 treatment groups.

### Serum thyroid hormone and TSH

Serum thyroid hormone and TSH levels of the three groups 3 months after initiation of the experiment are shown in Table 1. SCH and euthyroid in rats were confirmed by detecting serum FT3, FT4, and TSH levels. After 45 days of MMI administration, we detected increased levels of TSH (*P* <0.05) and normal levels of FT4 and FT3 in experimental rats compared with rats in the control group. The SCH rat models were successful. After 3 months, the SCH group showed higher TSH levels than did the control group (*P* <0.05), but no statistically significant difference in FT4 level between the SCH and control groups (*P* >0.05) was observed. After 45 days of L-T4 treatment, serum TSH concentration in the SCH+T4 treatment group, which approached that of the control group, was decreased compared with that in the SCH group (*P* >0.05). No significant differences in FT4 and FT3 were observed among the three groups (*P* >0.05).

**Table 1:** Serum thyroid hormone, TSH

	C	SCH	SCH+T4
FT3 (ng/ml)	0.87 ± 0.15	0.81 ± 0.12	0.83 ± 0.19
FT4 (ng/ml)	59.82 ± 9.56	52.31 ± 10.3	54.12 ± 9.43
TSH (μIU/ml)	0.95 ± 0.48	17.4 ± 6.41*	0.83 ± 0.29^#^

FT3: Free triiodothyronin; FT4: Free thyroxin; TSH: Thyroid-stimulating hormone; *P < 0.05 vs. control group; ^#^*P* < 0.05 vs. SCH group.

### Bone metabolism markers

The bone metabolism marker data of the three groups are shown in Table 2. The rats with SCH displayed decreased serum TRACP-5b and BALP concentrations compared with the control rats (*P* < 0.05). Conversely, the concentrations in the SCH+T4 treatment group, which approached those of the control group, were significantly higher than in the SCH group (*P* <0.05). However, no significant differences in serum Ca, P, and CTX levels were observed among the three groups (*P* > 0.05).

**Table 2:** Bone metabolism markers

	C	SCH	SCH+T4
Serum bone BALP (ug/l)	1.88 ± 0.11	1.41 ± 0.16*	1.57 ± 0.13^#^
Serum TRACP-5b (pg/ml)	334.48 ± 47.09	224.26 ± 53.70*	274.19 ± 49.70^#^
Serum CTX(ug/l)	0.30 ± 0.03	0.26 ± 0.04	0.29 ± 0.04
Serum Ca (mmol/l)	2.40 ± 0.10	2.26 ± 0.11	2.36 ± 0.09
Serum P (mmol/l)	1.51 ± 0.24	1.50 ± 0.29	1.47 ± 0.19

BALP: Bone alkaline phosphatase; TRACP-5b: Tartrate resistant acid phosphatase 5b; CTX: C-terminal telopeptide type I collagen; Ca: Calcium; P: Phosphorus; *P < 0.05 vs. control group; ^#^*P* < 0.05 vs. SCH group.

### Bone mineral density data

The BMD data of the three groups are shown in Table 3. The rats in the SCH group showed lower BMD in spine, upper limbs, lower extremities, trunk, pelvis, and average of whole body compared with rats in the control group (*P* < 0.05). However, no significant differences in BMD values in skull and ribs were observed between the SCH group and control group (*P* > 0.05). Compared with rats in the SCH group, the rats in SCH+T4 treatment group displayed lower pelvis BMD (*P* < 0.05). There was no significant difference in the BMD in other sites between the SCH group and the SCH+T4 treatment group (*P* > 0.05).

**Table 3:** Bone mineral density (BMD) data

	C	SCH	SCH+T4
skull (mg/cm^3^)	0.25 ± 0.04	0.24 ± 0.07	0.24 ± 0.04
Spine (mg/cm^3^)	0.21 ± 0.02	0.18 ± 0.02*	0.17 ± 0.01
Upper limb (mg/cm^3^)	0.21 ± 0.04	0.17 ± 0.06*	0.17 ± 0.02
Lower extremity (mg/cm^3^)	0.23 ± 0.07	0.17 ± 0.02*	0.17 ± 0.04
Trunk (mg/cm^3^)	0.15 ± 0.01	0.11 ± 0.01*	0.11 ± 0.04
Rib (mg/cm^3^)	0.20 ± 0.04	0.20 ± 0.01	0.19 ± 0.04
pelvis (mg/cm^3^)	0.23 ± 0.04	0.15 ± 0.02*	0.13 ± 0.06^#^
Whole body (mg/cm^3^)	0.22 ± 0.03	0.18 ± 0.01*	0.18 ± 0.03

*P < 0.05 vs. control group; ^#^*P* < 0.05 vs. SCH group.

### Biomechanical properties

The biomechanical data of the three groups are shown in Table 4. Maximum load, stiffness, Elastic modulus, and energy absorption were significantly decreased in the SCH group compared with the control group (*P* < 0.05). After 45 days of L-T4 treatment, rats in the SCH+T4 treatment group displayed an increase in maximum load and elastic modulus compared with the rats in the SCH group (*P* < 0.05). Increases in stiffness and energy absorption were observed in the SCH+T4 treatment group compared with the SCH group, but these increases were not significant (*P* > 0.05). No significant differences in these parameters were observed between the SCH+T4 treatment group and the control group (*P* > 0.05).

**Table 4:** Biomechanical properties data

	C	SCH	SCH+T4
Maximum load (N)	190.67 ± 20.18	132.59 ± 15.42*	163.83 ± 7.97^#^
Stiffness (N/mm)	179.65 ± 29.90	121.61 ± 16.61*	159.64 ± 8.67
Energy absorption (N*mm)	104.71 ± 14.81	70.59 ± 19.82*	89.24 ± 12.44
Elastic modulus (N/mm2)	10174.98 ± 1627.69	5601.41 ± 1753.84*	8779.3156 ± 2860.68^#^

*P < 0.05 vs. control group; ^#^*P* < 0.05 vs. SCH group.

### Bone histology

**Figure 2:**
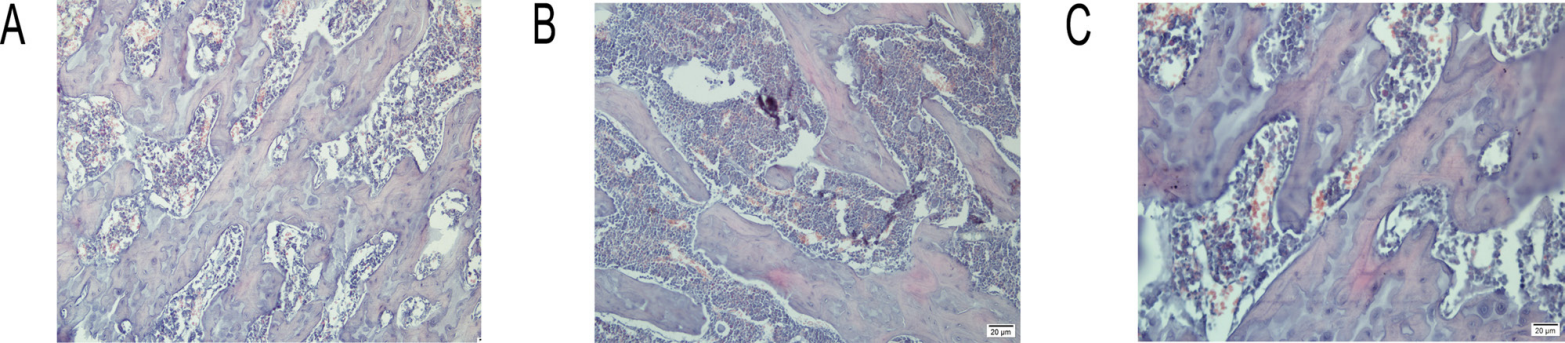
Histopathological examination in three groups Representative histological sections of the proximal femurs stained with H&E (40×). (**A**) Control group, (**B**) SCH group, and (**C**) SCH+T4 group.

Histopathological examination revealed increased disconnections and separation of the trabecular bone network, as well as the reduction of trabecular bone mass and number throughout the proximal metaphysis of the femur in the SCH group compared with the control group. The trabecular bone of the femur in rats with SCH presents a tip shape. Compared with rats with SCH, the quality of trabecular bones of rats in the SCH+T4 treatment group were improved with the increased number of bones and reduced separations in the trabecular bone network (Figure 2).

## DISCUSSION

Thyroid disorders are common endocrine diseases. Although overt thyroid dysfunction is a risk factor of secondary osteoporosis, the effects of SCH on bone are still under discussion [18–21].

In this study, SCH was induced in rats by MMI administration (10 mg/kg), once daily by gavage. The dose of MMI in our study was determined on the basis of previous studies [26–29]. These studies established a hypothyroid rat model, so we reduced the dose by fivefold to establish the SCH rat model. After 45 days of MMI administration, we detected increased levels of TSH (*P* < 0.05) and normal levels of FT4 and FT3 in experimental rats compared with rats in the control group. The SCH rat models were successful, and the chosen dose of MMI was effective for inducing SCH in the experimental groups. Our experiments were designed to investigate the effects of SCH on bone and to determine whether L-T4 administration causes changes in bone parameters by use of the SCH rat model. Therefore, we continued to give the rats MMI (10 mg/kg) once daily by gavage but only added a daily intragastric injection of L-T4 at the dose of 2 μg/kg [30, 31] in the SCH+L-T4 group and concurrently measured the thyroid hormone and TSH levels.

Our study focused on bone changes associated with SCH state. We selected BMD, bone metabolic markers, biomechanical properties, and bone microstructure as indicators of osteoporosis in rats. We have shown that rats with SCH have lower BMD in spine, upper limbs, lower extremities, trunk, pelvis, and whole body. However, no significant difference was observed in skull and ribs between the SCH group and the control group. A possible reason is the different component ratio of cortical and cancellous bone between flat bone and long bone. This development needs further confirmation. In rats with SCH, the analysis of bone metabolic markers revealed a decreased bone turnover.

We also performed biomechanical analysis of bone, which provides important predictors of fracture risk [32] and noted that biomechanical properties of bone tissue in rats with SCH differ from properties in control rats. These data, together with the lower trabecular number in rats with SCH than in control rats, demonstrated decreased bone mass and strength in the SCH group. In our study, alterations in decreased BMD and bone metabolic marker levels in the SCH state coincided with reduced trabecular number and biomechanical properties. These data confirm that SCH has destructive effects in bone and might increase the risk of osteoporosis, which could be the result of direct effects of TSH on bone metabolism [33].

TSH, which suppresses skeletal remodeling [12], not only inhibits osteoblastic bone formation but also inhibits osteoclastogenesis[34, 35]. The thyroid hormone levels in rats with SCH were found to be normal. Therefore, we can speculate that a high TSH level in SCH has a stronger inhibition effect on bone formation than on bone resorption, which destroys the balance of bone metabolism, resulting in a reduction of bone mass and strength. Peter et al. [9] found an increased risk of fractures in untreated hypothyroidism patients beginning up to 8 years before the diagnosis of clinical hypothyroidism in a nationwide follow-up study. However, most overt hypothyroidism patients would have signs or symptoms of clinical hypothyroidism earlier than 8 years. Thus, this finding suggests that these hypothyroidism patients have SCH, which could alter their bone metabolism and increase fracture risk. However, the studies on endogenous SCH and BMD or fracture risk have produced controversial results. Liang et al. [36] reported that the incidence of bone mass loss and lower levels of serum Ca^2+^ in SCH patients increases compared with the incidence in euthyroid populations. Nagata et al. [37] found that SCH correlates with the lower quantitative heal ultrasound (QUS) used to assess bone structure in postmenopausal women, and results of this study suggest that elevation of serum TSH concentration in SCH alters bone structure but not bone turnover. Similarly, Lee et al. [18] and Bertoli et al. [38] reported that women with SCH have reduced femoral neck and leg BMD. Two prospective studies have shown increased risk of hip fracture in both men and women with SCH [19, 39]. However, Kim et al. [6] found that SCH is not associated with reduced bone health, including BMD [40–42] and bone turnover markers. Other studies and meta-analyses found no association between SCH and fracture risk or BMD. These differences could be the result of the diversity of populations as well as the different scale of research.

In the present study, the SCH state is reversed after L-T4 treatment. We also used a complete set of investigative modalities to examine bone response to L-T4 treatment and confirmed that short-term L-T4 has osteoprotective effects, at least in the SCH rat model. The levels of serum bone metabolic markers, which are widely used to measure the effects of therapeutic modalities on bone remodeling [43] were increased in rats with SCH after 45 days of L-T4 treatment. However, no changes in the mean values of BMD were observed after L-T4 treatment. The reason for these results might be that bone metabolic markers can sensitively reflect the near-term change in bone metabolism, so changes in serum TRACP-5b and BALP appear earlier than changes in BMD. We also observed that the femurs of rats with SCH receiving L-T4 resisted deformation much better than femurs of the SCH group and showed increased biomechanical properties. This finding indicates that L-T4 treatment maintained bone strength and protected against the deleterious effects of SCH. Consistent with these changes, we observed an increase in trabecular number of L-T4 treated rats during pathology examination. Such biomechanical and structural improvements suggest that L-T4 treatment results in enhanced bone quality.

L-T4 is the treatment for patients with hypothyroidism [44], who often require lifelong thyroid hormone therapy, and L-T4 is also used to treat subclinical hypothyroidism [45]. The proper amount of L-T4 increases serum FT4 and FT3 levels and reduces TSH secretion, which has a negative effect on the pituitary in hypothyroidism [46]. Studies have demonstrated that treatment with L-T4 is effective in improving alterations in patients with SCH, such as cardiovascular function [47]. However, L-T4 replacement therapy in bones has produced controversial results. Park et al. [48] confirmed that in osteoblast-like cells, L-4 induces bone formation, differentiation, and mineralization by increasing the expression of angiopoietin 1. This finding suggests that L-4 is a potential bone production agent. However, Pines et al. [49] reported that L-4 prevented the beneficial effect of hormone-replacement therapy on lumbar spine BMD in postmenopausal women with SCH. Similarly, Vignera et al. [50] observed a reduction of the lumbar vertebrae BMD after 1 year of a fixed dose of L-T4 for the treatment of nodular thyroid disease, which was associated with increased serum alkaline phosphatase level and urinary excretion of hydroxyproline. A meta-analysis [51] resulted in the conclusion that replacement therapy with L-T4 correlates with bone loss in the spine and hip in premenopausal women. However, some studies demonstrated that long-term L-T4 therapy does not cause changes to skeleton and has no adverse effect on BMD or bone turnover [52, 53]. Although whether patients with SCH should be treated is controversial, one benefit of L-4 therapy is prevention of hypothyroidism [44]. Thus, the most prevalent trend is that patients with initial TSH > 10 mIU/L, with symptoms of hypothyroidism and TSH levels between 5 and 10 mIU/L, should be treated [44].

In conclusion, our data demonstrated that rats with SCH have decreased BMD, serum bone metabolic markers, biomechanical properties, and bone microstructure. The high level of TSH in SCH is a factor in these changes. L-T4 treatment inhibits these changes to some extent. The current evidence should be confirmed in larger trials and the impact of L-T4 on other osteoporosis risk factors should be explored. Further mechanisms should be investigated to explain the function of SCH in osteoporosis.
